# Efficacy of a thermoplastic mask and pneumatic abdominal compression device for immobilization in stereotactic ablative radiotherapy of spine metastases

**DOI:** 10.1002/acm2.14577

**Published:** 2024-12-01

**Authors:** Yohan A. Walter, Daniel B. Speir, William E. Burrell, Chiachien J. Wang, Hsinshun T. Wu

**Affiliations:** ^1^ Department of Radiation Oncology Willis Knighton Cancer Center Shreveport Louisiana USA

**Keywords:** immobilization, motion management, SABR, SBRT, spine metastases, stereotactic ablative radiotherapy, stereotactic body radiation therapy, treatment planning

## Abstract

Stereotactic ablative radiotherapy (SABR) has become a key technique in management of spine metastases. With improved control over treatment plan dosimetry, there is a greater need for accurate patient positioning to guarantee agreement between the treatment plan and delivered dose. With serious potential complications such as fracture and myelopathy, the margins of error in SABR of the spine are minimal. In this study, we assessed the performance of two patient immobilization setups in SABR for spinal metastases. First, a Type‐S head and shoulders mask (CQ Medical, Avondale, PA), and second, the BPL1 setup, which includes a wing board, vacuum bag, and the Respiratory Belt for the Body Pro‐Lok ONE (CQ Medical, Avondale, PA). Immobilization was assessed using image‐guided intrafraction repositioning shifts. Required planning target volume (PTV) margins were calculated based on repositioning data for 172 treated fractions using 2 standard deviation (2SD) and analytic approaches. Overall, 91.7% and 74.1% of fractions treated had total 3D repositioning shifts ≤3.0  mm using the Type‐S and BPL1 setups, respectively. In the thoracic spine, 43.2% and 46.5% of fractions had shifts ≤1.5  mm for the respective setups. Suggested margins were under 3.5  mm in all directions and use cases. In the posterior‐anterior direction, the BPL1 setup had a 0.6  mm smaller suggested margin for the thoracic spine compared to the Type‐S setup, at 1.4  mm, calculated using the analytic approach. Both the Type‐S and BPL1 setups are effective for immobilization in spine SABR. The Type‐S demonstrated superior immobilization in the upper spine and remains the clinical standard for cervical and upper thoracic spine positioning. The BPL1 setup showed effective immobilization in use cases treating the mid‐to lower thoracic spine and lumbar spine and remains our clinical standard for those use cases. Results additionally demonstrate feasibility of potential PTV margin reduction.

## 1 INTRODUCTION

With modern advances in radiation treatment delivery, stereotactic ablative radiotherapy (SABR), or stereotactic body radiation therapy (SBRT) has proven safe and effective in management of spine metastases.^1^, ^2^, ^3^, ^4^, ^5^, ^6^ However, the proximity of targets to the spinal cord or cauda equina and the compromised structure of vertebrae with active lesions bring potential for critical toxicities, such as radiation myelitis and bone fracture.^1^, ^2^, ^4^, ^7^, ^8^ Though overall, adhering to established spinal cord dose tolerances correlates with reduced toxicities,^4^ advancements in patient positioning have crucially improved agreement between delivered and planned doses.^9^

Today, available immobilization and patient positioning techniques are numerous and wide in variety. Though this provides improved potential customization in patient motion management, it is neither feasible nor simple for most centers to perform in‐house evaluations of all available techniques. Furthermore, excessive variety can complicate the clinical decision‐making process when developing patient‐specific motion management strategies. Additionally, complex patient setups can extend treatment times, which can negatively affect patient tolerance and throughput.

Therefore, when developing clinical protocols for patient immobilization in SABR for spine metastases, it is essential to consider balancing simplicity of the setup with its efficacy in maintaining an acceptable patient position.

In our clinic, most spine SABR cases are immobilized using either a Type‐S head‐and‐shoulders thermoplastic mask (CQ Medical, Avondale, PA, USA), or a simple setup using a wing board, vacuum bag, and the Respiratory Belt for the Body Pro‐Lok ONE system (BPL1, CQ Medical, Avondale, PA, USA).

Generally, thermoplastic masks like the Type‐S are regarded as the clinical standard for immobilization in SABR of the cervical and upper thoracic spine.^9^ However, as the Type‐S mask achieves its superior results with primary anchor points in the head and shoulders, the larger distance between anchor points and targets in the mid‐thoracic spine or inferior may result in less rigid immobilization near distal target areas. Furthermore, a recent dosimetric study by Okamoto and colleagues correlated density shifts along the radiological path length due to respiratory motion in the liver with measured discrepancies in target and spinal cord doses from the nominal treatment plan.^10^ Therefore, both improved immobilization and respiratory motion management may be merited for targets in the lower thoracic spine.

For cases treating the mid‐thoracic spine and lower, we thus use the versatile BPL1setup for patient immobilization. The belt doubles as an immobilization device and as an abdominal compression device, limiting respiratory motion via the forced shallow breathing technique. Though the impact of abdominal compression on respiratory motion has been studied extensively,^11^, ^12^, ^13^, ^14^, ^15^ to our knowledge, the efficacy of compression belts as immobilization devices for SABR of the spine has yet to be evaluated.

The purpose of this study was thus twofold: first, to assess the efficacy of the widely used Type‐S mask and the flexible BPL1 setup in immobilization for SABR of the spine, and second, to develop recommendations for standard setup margins based on the pooled positioning data.

## 2 METHODS

### 2.1 Patient cohort

In this IRB‐exempt study, intrafraction repositioning data were collected for 50 patients undergoing SABR for spine metastases at Willis Knighton Cancer Center (Shreveport, LA, USA) in 2016–2024. In total, 51 courses treated in 172 fractions were analyzed. Sixteen fractions treated metastases in the cervical spine, 113 fractions treated the thoracic spine, and 43 treated the lumbar spine.

### 2.2 Setup and treatment delivery

**FIGURE 1:**
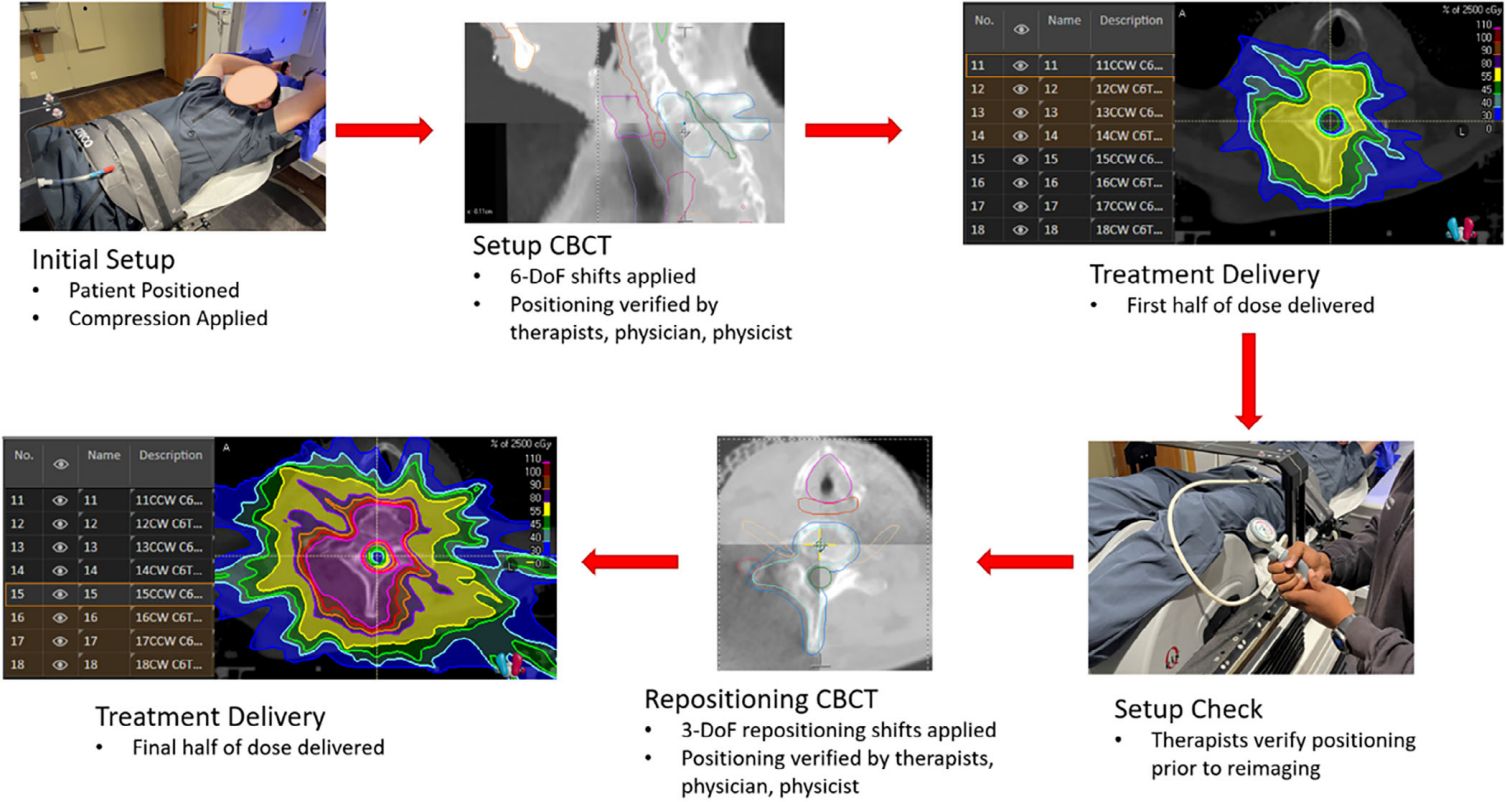
SABR treatment workflow demonstrating the symmetric dose repainting technique. DoF = Degree of Freedom.

All patients included in this study were immobilized with either a Type‐S Fibreplast 3.2  mm Variable Perf Head & Shoulder mask with Integrated Shims (*N *= 60 fractions) or a simple setup using a wing board, Vac‐Lok bag (CQ Medical, Avondale, PA, USA), and the Body Pro‐Lok ONE Respiratory Belt (Figure 1, *N =* 112 fractions). In both setups, a knee wedge (SofTouch, CQ Medical, Avondale, PA, USA) was added for comfort (Figure 1).

Treatments were delivered in 1–5 fractions on an Elekta VersaHD linear accelerator (Elekta AB, Stockholm, Sweden). Plans used volumetric modulated arc therapy (VMAT) delivery and the dose repainting technique, in which the beams were duplicated, and MU scaled by 50%, creating two symmetric dose halves. Dose repainting facilitated intrafraction repositioning halfway through treatment delivery with minimal dose perturbation. Treatments were delivered in four to eight total arcs.

Between symmetric dose halves, intrafraction cone beam CT (CBCT) was used for image‐guided repositioning (Figure 1). Intrafraction CBCT images were aligned to the reference CT via 3‐degree‐of‐freedom (3‐DoF) automatic rigid registration in the Elekta system. All registration results were reviewed by two therapists, a physician, and a physicist prior to beam delivery. These corrections were applied and recorded for analysis in this study. Shifts were recorded as executed patient movements. The total 3D repositioning shift was defined as the vector addition of the directional shifts. For the cases included in this study, the time between initial image‐guided positioning and intrafraction repositioning averaged 7.7 ± 2.8 min.

### 2.3 PTV margin calculation

The suggested setup margin was determined using two techniques. First, using two standard deviations (2SD), which corresponds with 95% of treatments having intrafraction positional errors within the suggested margins, and second, an extension of the van Herk margin recipe,^16^ as described by Janssen et al.^17^ The extended van Herk (EvH) recipe factors the number of fractions, *N*, into the margin requirement.

(1) $$\mathrm{PTV}\;\mathrm{Margin}=2.5\Sigma_{N}+0.7\sigma_{N}$$

(2) $$\Sigma_{N}^{2}=\frac{1}{N}\;\sigma^{2}$$

(3) $$\sigma_{N}^{2}=\frac{N-1}{N}\;\sigma^{2}$$

The recipe uses standard deviations of systematic (*∑*) and random (*σ*) components of error which are specific to each workflow and disease site. This analytic method calculates planning target volume (PTV) expansions such that the clinical target volume (CTV) is covered by the 95% isodose line in 90% of fractions.^16^, ^17^

Margins were calculated for 3‐fraction and 5‐fraction schemes with intrafraction repositioning, as these were the most common schedules used in this study. *N* = 6 and *N* = 10 were used for 3‐and 5‐fraction regimens, respectively, to simulate executed intrafraction repositioning halfway through treatment. Uncertainties used for calculations are listed in the supplementary material (Table S1).

### 2.4 Data analysis

Data analysis was performed using analysis of variance (ANOVA) in OriginPro (OriginLab, Northampton, MA, USA). *p* < 0.05 indicated statistically significant differences between means.

## 3 RESULTS

### 3.1 Repositioning shifts

Table 1 shows the percentage of fractions with total 3D repositioning shifts less than or equal to 1.5 mm, 3.0 mm, and 5.0 mm. The Type‐S setup achieved higher rates of intrafraction shifts under the chosen thresholds, except thoracic spine immobilization at the 1.5 mm threshold. In the posterior‐anterior (P/A) direction, 100.0% of fractions and 99.1% of fractions had shifts ≤3.0 mm for the Type‐S and BPL1 setups, respectively. 83.3% and 91.5% of fractions had P/A shifts ≤1.5 mm, respectively.

**TABLE 1:** Percentage of fractions with 3D repositioning shifts less than or equal to clinically relevant thresholds.

Type‐S, % of 3D shifts				BPL1, % of 3D shifts			
Region (# Fx)	≤1.5 mm	≤3.0 mm	≤5.0 mm	Region (# Fx)	≤1.5 mm	≤3.0 mm	≤5.0 mm
All (*N* = 60)	50.0	91.7	100.0	All (*N* = 112)	46.4	74.1	97.3
Cervical (*N* = 16)	68.8	100.0	100.0	Cervical (*N* = 0)	–	–	–
Thoracic, T1‐T4 (*N* = 44)	43.2	88.6	100.0	Thoracic, T5‐T12 (*N* = 69)	46.5	74.4	95.3
Lumbar (*N* = 0)	–	–	–	Lumbar (*N* = 43)	46.4	73.9	98.6

Abbreviation: BPL1, Body Pro‐Lok ONE setup.

**FIGURE 2:**
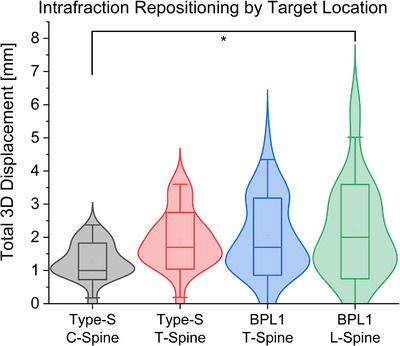
Plot of the measured 3D intrafraction repositioning shifts for treated fractions by target location and setup. 3D shifts were calculated as the vector addition of individual directional shifts. Box widths are ±1 standard deviation, whiskers extend to ±2SD. BPL1 = Body Pro‐Lok ONE setup.

Overall, treatments in the cervical spine with the Type‐S setup had the smallest 3D displacement in this study. These cases also had the smallest standard deviation in shift magnitude (Figure 2, Table 2). However, the only statistically significant difference in magnitude was observed between cases using the Type‐S setup in the cervical spine and the BPL1 setup immobilizing the lumbar spine (*p* < 0.05).

**TABLE 2:** Summary of directional 3D repositioning data by target location and setup.

Type‐S Repositioning shifts (mm) (±)						BPL1 Repositioning shifts (mm) (±)				
Region (# Fx)		I/S	R/L	P/A	3D	Region (# Fx)	I/S	R/L	P/A	3D
All (*N* = 60)	Avg	0.6	−0.1	−0.1	1.7	All (*N* = 117)	0.1	0.4	0.2	2.1
	SD	0.9	1.2	1.1	0.8		1.6	1.6	1.0	1.3
Cervical (*N* = 16)	Avg	0.1	−0.2	−0.3	1.3	Cervical (*N *= 0)	–	–	–	–
	SD	0.8	0.8	0.8	0.6		–	–	–	–
Thoracic, T1‐T4 (*N* = 44)	Avg	0.8	−0.1	0.0	1.9	Thoracic, T5‐T12 (*N* = 69)	0.2	0.0	0.3	2.0
	SD	0.9	1.3	1.2	0.9		1.6	1.5	0.8	1.2
Lumbar (*N* = 0)	Avg	–	–	–	–	Lumbar (*N* = 43)	−0.1	0.7	0.1	2.2
	SD	–	–	–	–		1.4	1.6	1.3	1.4

Abbreviations: I/S, inferior‐superior direction, R/L, right‐left direction; P/A, posterior‐anterior direction; SD, standard deviation.

### 3.2 PTV margin requirements

Calculated setup margins based on the 95^th^ percentile and using the EvH method are listed in Table 3 for each direction. Across all cases, the BPL1 setup required a larger setup margin in the superior‐inferior and right‐left directions as compared to the Type‐S setup. However, the BPL1 setup required a reduced margin in the A/P direction for the thoracic spine as compared to the mask setup.

**TABLE 3:** Directional setup margins calculated from pooled data.

	Type‐S setup margins (mm)			BPL1 setup margins (mm)			
Region	I/S	R/L	P/A	I/S	R/L	P/A	Calculation method
All	1.8	2.3	2.2	3.1	3.1	2.0	2SD
	1.8	2.1	2.1	2.8	2.7	1.9	EvH, *N *= 6
	1.6	1.9	1.8	2.4	2.4	1.7	EvH, *N* = 10
Cervical	1.5	1.7	1.6	–	–	–	2SD
	1.6	1.6	1.6	–	–	–	EvH, *N* = 6
	1.4	1.4	1.4	–	–	–	EvH, *N *= 10
Thoracic	1.8	2.6	2.4	3.3	2.9	1.5	2SD
	1.8	2.3	2.2	2.9	2.6	1.6	EvH, *N *= 6
	1.5	2.0	1.9	2.5	2.3	1.4	EvH, *N* = 10
Lumbar	–	–	–	2.9	3.1	2.7	2SD
	–	–	–	2.6	2.7	2.4	EvH, *N *= 6
	–	–	–	2.3	2.4	2.1	EvH, *N *= 10

Abbreviations: 2SD, 2 standard deviations; EvH, extended van Herk recipe.

Simulating dose repainting using *N* = 6 and *N *= 10 reduced margin requirements by 0.5 ± 0.1 mm and 0.4 ± 0.1 mm for 3‐and 5‐fraction regimens, respectively, averaged across all sections of the vertebral column (Table 3 & S2). The 2SD method yielded 0.2 ± 0.2 mm and 0.4 ± 0.2 mm larger margins than the EvH method, using *N* = 6 and *N* = 10, respectively.

## 4 DISCUSSION

Today, intensity‐modulated delivery in SABR has allowed users to treat vertebrae while aggressively shaping dose to avoid the spinal cord. Though superior control has allowed for greater dose escalation to targets and dose reduction to the spinal cord, this has also placed greater demand on delivery accuracy to ensure agreement between delivered dose and the nominal treatment plan. Additionally, increasingly effective immobilization can reduce the required setup margins used in planning, which can reduce the necessary dose to organs at risk. Patient positioning and immobilization strategies are thus critical for ensuring safe and efficacious delivery.

In our clinic, the Type‐S mask and the BPL1 setup present simple, yet effective means of achieving positioning within acceptable margins. The Type‐S mask demonstrated superior overall immobilization in the cervical spine, which agrees with data reported in previous studies.^9^, ^18^

Though the Type‐S mask generally required smaller 3D repositioning shifts than the BPL1 setup, the results may have been influenced in part by the clinical use cases. In our clinic, the Type‐S mask was generally reserved for treatments in the cervical and upper thoracic spine (T1‐T4), whereas the BPL1 setup was used for all other cases. The lack of data for treatments in the same regions for both setups was thus a limitation of this study.

Aside from total 3D repositioning shifts, when comparing the Type‐S mask to the BPL1 setup for all cases, the most significant differences in standard deviations were in the inferior‐superior (I/S) and right‐left (R/L) directions. In the P/A direction, the spread in data was remarkably similar between the two setups, with the BPL1 having a slightly smaller SD. These differences can be attributed in part to immobilization anchor points for each setup. The Type‐S mask has several rigid anchor points on the head and shoulders, which limits both lateral and longitudinal movement, whereas the primary anchor point for the BPL1 setup is the upper abdomen, which is under compression between the belt and treatment table. The BPL1 setup may thus better limit P/A motion as compared to other directions.

Considering the positional accuracy needs in SABR of the spine, any uncertainty is suboptimal. However, most dosimetrically critical are immobilization in the anterior‐posterior and right‐left directions, which directly affect delivered dose to the spinal cord. Based on our collected data, both setups require margins under 2.5 mm in the A/P direction and 3.5 mm in all other directions using both the 2SD and EvH calculation methods. In the thoracic spine, both setups required a margin under 3.0 mm in all directions, and the BPL1 setup required the smallest margin in this study at 1.4 mm in the A/P direction, 0.6 mm smaller than for the Type‐S setup using the EvH method (*N* = 10 fractions). Therefore, though the BPL1 setup has greater positional uncertainty in the I/S direction, its performance in the thoracic spine is comparable to that of the Type‐S mask, demonstrating the feasibility and efficacy of the BPL1 setup for immobilizing the spine.

The limitations of various PTV margin calculation methods have been covered extensively in literature.^16^, ^17^, ^19^, ^20^ The analytic EvH approach gave a rigorous determination of margins for our workflow. However, the errors used in the calculations were determined through internal studies and those reported in literature for our equipment.^21^, ^22^ Therefore, the EvH calculations presented here may not be directly applicable to other clinics. The 2SD approach gives a more generalizable estimate, though key factors are excluded from the calculation, including image registration uncertainties and intrafraction repositioning. Additionally, since the margins were determined based on CBCT‐guided intrafraction repositioning shifts, PTV margins were calculated with the assumption that patient motion was a discrete and instantaneous process. Though Janssen et al. proposed a model for continuous motion in margin calculations,^17^ the method was not used in this study. Both calculation methods therefore likely yielded conservative estimates of margin requirements.

Overall, the two setups present simple, convenient options for immobilization of the spine. Long thermoplastic masks are regarded as the clinical standard for cases treating the head and neck regions, while a variety of immobilization systems are available for immobilization in the thorax and abdomen. The BPL1 setup used in this study achieves a valuable combination of rigidity, customizability, and economy, where all elements of the setup can be customized to meet patient needs and are reusable. Alternatively, masks are typically discarded after use for each patient.

## 5 CONCLUSION

In this study, we have demonstrated the efficacy of the Type‐S long thermoplastic mask and a simple BPL1 setup in immobilization of the spine. Based on our clinical data, both setups meet the steep positional requirements of spine SABR, while providing ease of use. In general, the Type‐S mask showed superior results for immobilization of the cervical spine and is our clinical standard for immobilization in the head and neck region. However, the BPL1 showed high performance in the thoracic and lumbar spine, which, combined with its flexibility and reduced waste, has made the BPL1 setup the favored strategy for treatments in or below the upper thoracic spine.

Future studies are warranted to determine the dosimetric impact of the reduced planning margins calculated from our clinical data. However, a formal risk assessment may be necessary prior to adopting suggested margins, as shrinking PTV margins carries the increased risk of underdosing targets.

## AUTHOR CONTRIBUTIONS

All authors made substantial contributions to the design, preparation, and execution of the presented study, including data acquisition, analysis, interpretation, manuscript drafting, and review.

## CONFLICT OF INTEREST STATEMENT

YW, DS, CJW, and HTW report active research agreement with CQ Medical (Avondale, PA, USA). YW and DS declare receiving travel funding from CQ Medical.

## Supporting information



Supporting Information

## Data Availability

The data that support the findings of this study are available from the corresponding author upon reasonable request.
