# Supplementary material for: Efficacy of a thermoplastic mask and pneumatic abdominal compression device for immobilization in stereotactic ablative radiotherapy of spine metastases
Source: J Appl Clin Med Phys. 2024 Dec 1;26(4):e14577. doi: 10.1002/acm2.14577 (PMC11969103; doi:10.1002/acm2.14577)
Supplement: Supplementary file 1 — Supporting Information [file ACM2-26-e14577-s001.docx]

**Supplement Material for manuscript, “Efficacy of a Thermoplastic Mask and Pneumatic Abdominal Compression Device for Immobilization in Stereotactic Ablative Radiotherapy of Spine Metastases”**

Table S1: Summary of uncertainties used in planning target volume margin calculations.

| **Error Source** | **SD, *σ*, I/S [mm]** | **SD, *σ*, R/L [mm]** | **SD, *σ*, P/A [mm]** |
| --- | --- | --- | --- |
| Image Registration^1,2^ | 0.5 | 0.5 | 0.5 |
| Mechanical Alignment | 0.3 | 0.2 | 0.3 |
| Target Positioning | Measured SD | Measured SD | Measured SD |

Abbreviations: SD = standard deviation, I/S = inferior-superior direction, R/L = right-left direction, P/A = posterior-anterior direction

Table S1 shows the uncertainties used in the calculation of PTV margin requirements with the extended van Herk approach. Image registration uncertainties were reported in literature for the Elekta XVI system.^1,2^ The standard deviation for mechanical isocentricity was measured in an internal study using routine quality assurance data over a five-year period. Patient positioning uncertainty was measured in this study.

Table S2: Extended van Herk margin calculations without repositioning (N=3 and N=5).

|  | Type-S Setup Margins [mm] | | | BPL1 Setup Margins [mm] | | | Calculation Method |
| --- | --- | --- | --- | --- | --- | --- | --- |
| Region | I/S | R/L | P/A | I/S | R/L | P/A |  |
| All | 2.2 | 2.6 | 2.5 | 3.4 | 3.3 | 2.4 | EvH, N=3 |
|  | 1.9 | 2.2 | 2.2 | 2.9 | 2.8 | 2.0 | EvH, N=5 |
| Cervical | 1.9 | 2.0 | 2.0 | -- | -- | -- | EvH, N=3 |
|  | 1.7 | 1.7 | 1.7 | -- | -- | -- | EvH, N=5 |
| Thoracic | 2.1 | 2.8 | 2.7 | 3.5 | 3.1 | 1.9 | EvH, N=3 |
|  | 1.8 | 2.4 | 2.3 | 3.0 | 2.7 | 1.7 | EvH, N=5 |
| Lumbar | -- | -- | -- | 3.1 | 3.3 | 2.9 | EvH, N=3 |
|  | -- | -- | -- | 2.7 | 2.9 | 2.5 | EvH, N=5 |

Abbreviation: 2SD = 2 standard deviations, EvH = extended van Herk recipe.

Table S2 shows the calculated margins using the EvH method, simulating no executed repositioning. The margin requirements were unanimously larger than for calculations with simulated repositioning; however, this model carries the assumption that the SD of target positioning is the same for the entire fraction as it is at the halfway point where imaging was performed.

References

1. Sykes JR, Brettle DS, Magee DR, Thwaites DI. Investigation of uncertainties in image registration of cone beam CT to CT on an image-guided radiotherapy system. *Phys Med Biol*. 2009;54(24). doi:10.1088/0031-9155/54/24/002
2. Barber J, Sykes JR, Holloway L, Thwaites DI. Automatic image registration performance for two different CBCT systems; Variation with imaging dose. In: *Journal of Physics: Conference Series*. Vol 489. ; 2014. doi:10.1088/1742-6596/489/1/012070
